# Detection of large expansions in myotonic dystrophy type 1 using triplet primed PCR

**DOI:** 10.3389/fgene.2014.00094

**Published:** 2014-04-24

**Authors:** Susmita Singh, Amy Zhang, Stephen Dlouhy, Shaochun Bai

**Affiliations:** Department of Medical and Molecular Genetics, Indiana University School of MedicineIndianapolis, IN, USA

**Keywords:** myotonic dystrophy type 1 (DM1), trinucleotide repeat, CTG, allelic expansion, triplet-repeat primed PCR

## Abstract

Myotonic dystrophy type 1 (DM1) is an autosomal dominant neuromuscular disease caused by expansion of a CTG trinucleotide repeat in the *DMPK* gene. Methodology for genetic testing of DM1 is currently not optimal, in particular for the early-onset patients in pediatric populations where large expanded (CTG)n alleles are usually common. Individuals who are homozygous for a normal allele and individuals who are heterozygous for one normal and one large expanded allele are indistinguishable by conventional PCR, as both generate a single product of the normal allele. Thus, reflex Southern blot has often been needed to distinguish these cases. With the aim to decrease the need for reflex Southern blot tests, a novel, single-tube CTG repeat primed PCR technology was designed to distinguish the true homozygous patients from the individuals whose large alleles are missed by conventional PCR. The method utilizes two gene-specific primers that flank the triplet repeat region and a third primer set complementary to the repeated region to detect the large alleles. Compared to traditional PCR, this novel Triplet-repeat Primed PCR can detect the presence of large expanded alleles with demonstrating a ladder pattern. Using this single-step protocol, 45 specimens were tested. The alleles with sizes~í~85 repeats were determined by the gene specific primers. 13 abnormal alleles, which were missed by conventional PCR, were successfully detected by the Triplet-repeat Primed PCR. All the abnormal alleles were confirmed and measured by Southern Blot analysis. In summary, optimized Triplet-Primed PCR (TP-PCR) can accurately detect the presence of the large expanded alleles. With the ability to distinguish the true homozygous patients from the false negative homozygous individuals, the application of the optimized TP-PCR can significantly reduce the need of Southern Blot tests.

## INTRODUCTION

Mytonic dystrophy type 1(DM1) is the most common adult onset neuromuscular disorder, with a prevalence frequency ranging from 1 to 15 in 100,000, and is inherited in an autosomal dominant pattern (Turner and Hilton-Jones, 2010). DM1 is a multisystem disorder that widely effects skeletal and smooth muscle, the endocrine system, the eye, heart and central nervous system (Johnson and Heatwole, 2012). The clinical indications span from mild to severe. *DMPK* is the only reported gene in which mutations cause DM1. *DMPK* is located on chromosome 19q13.3, and encodes a serine-threonine kinase. The disease causing mutation is an unstable CTG triplet expansion in the 3′-untranslated region of the *DMPK* gene (Prior, 2009). In normal individuals, the number of CTG repeats ranges from 5 to 34. Individuals with 35 to 49 CTG repeats have not been reported to develop DM1, but the number of the CTG repeats may increase when this allele is passed on to the next generation. People are affected with DM1 if the number of CTG repeats is equal to or greater than 50 (Bird, 2013). The severity of symptoms in DM1 is correlated with the repeat size. Patients with congenital or severe DM1 tend to have thousand(s) of CTG repeats in the 3′UTR region of the *DMPK* gene (Zeesman et al., 2002).

The molecular diagnostic analysis of DM1 is to determine the number of CTG repeats in two alleles with fragment-length based techniques, such as PCR or Southern Blot (Prior, 2009). Conventional PCR can detect a lower range of DM1 expansions, thus covering the normal and premutated alleles. Optimized PCR conditions can detect full penetrance alleles with a size up to 85 CTG repeats. Detection of the larger expansions still has relied on Southern Blot. Compared to PCR-based clinical laboratory techniques, Southern Blot has drawbacks. The requirement of large amounts of DNA and use of radioactive materials makes this procedure time consuming while demonstrating low sensitivity. In order to develop an efficient and sensitive molecular method, Triplet-Primed PCR (TP-PCR) was first introduced for detection of large CTG repeat expansions by Warner et al. (1996). TP-PCR provides an advantage in rapidly identifying the large pathogenic CTG repeats. The aims of this study were to verify the validity of TP-PCR for the diagnosis of patients presenting with DM1 clinical findings and to simplify the testing procedure.

## MATERIALS AND METHODS

### STUDY SUBJECTS

A total of 45 samples were retrospectively analyzed, including 19 patients and 26 proficiency testing survey (PTS) samples. The patients were selected from previous clinical samples from 2008 to 2013. The PTS samples were selected from 2008 to 2013. The sample population represents a range of CTG sizes covering normal, mild abnormal, and classic mutants. An IRB protocol (protocol # 1401263105) was approved by the Human Subjects Office at Indiana University.

### CONVENTIONAL PCR

Genomic DNA was extracted from peripheral blood using the DNeasy Blood & Tissue Kit (Qiagen, Germantown, MD, USA). Conventional PCR was performed with 100 ng of genomic DNA using gene specific primers flanking the *DMPK* CTG repeat (Forward: DM101 5′ FAM-CTT CCC AGG CCT GCA GTT TGC CCA TC 3′ and Reverse: DM102 5′ GAA CGG GGC TCG AAG GGT CCT TGT AGC 3′; Gharehbaghi-Schnell et al., 1998). All normal homozygotes and expanded alleles were confirmed with Southern Blot.

### TRIPLET-REPEAT PRIMED PCR

**FIGURE 1:**
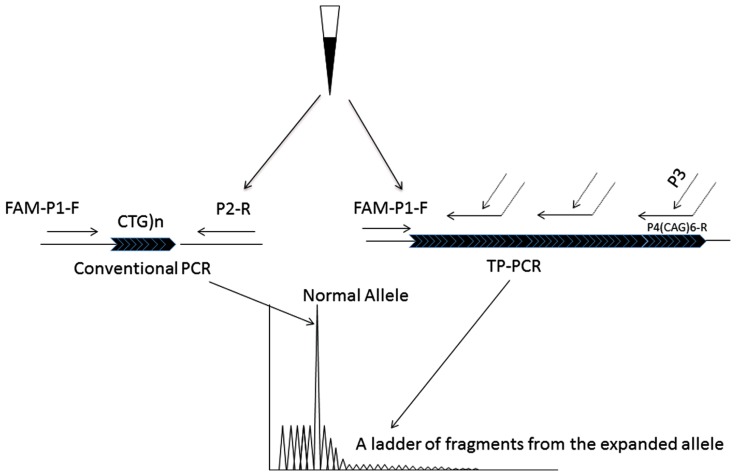
**The principle of the modified TP-PCR**. Two PCR procedures, conventional PCR and TP-PCR are simultaneously carried out. The conventional PCR primers, P1-F and P2-R bind to the gene specific regions that closely flank the CTG repeat, generating the smaller alleles. Primer P4(CTG)_6_-R has two parts: 6 CAG trinucleotides at the 3′-end sequence and an artificial sequence with no homology to the human genome at the 5′-end sequence. By the triplet priming from its 3′-end sequence, a ladder of PCR fragments differing by 1 CTG unit is produced.

Compared to the reported two-step procedure TP-PCR, in our study the triplet primed PCR was modified to a single-tube PCR reaction. The modified TP-PCR was performed with 100 ng of genomic DNA in a reaction volume of 25 μl. FAM-P1-Forward and P2-Reverse are designed as the gene specific PCR primers flanking the CTG repeat region (FAM-P1-Forward: 5′FAM-GGG-GCT-CGA-AGG-GTC-CTT-GT-3′ and P2-Reverse: 5′-GTG-CGT-GGA-GGA-TGG-AAC-ACG-3′). The forward primer was labeled with FAM fluorescence. Primer P4-(CAG)_6_-Reverse consists of two parts: a 3′-end with 6 CAG repeats and a 5′-end containing a universal sequence [P4-(CAG)_6_-Reverse: 5′AGC-GGA-TAA-CAA-TTT-CAC-ACA-GGA-CAG-CAG-CAG-CAG-CAG-CAG-3′]. P4-(CAG)_6_-Reverse can anneal to random complementary regions of the CTG track within the *DMPK* gene (**Figure 1**). The universal tail does not represent any homology to the human genome. P3 was designed to bind to the complement of the tail of the P4-(CAG)_6_-Reverse (P3: 5′AGC-GGA-TAA-CAA-TTT-CAC-ACA-GGA- 3′). The combination of primers was prepared in a ratio as: FAM-P1-Forward: P4-(CAG)_6_-Reverse:P3:P2-Reverse = 1.5:1:1.5:1.5, with a final working concentration of 0.6 μM:0.4 μM:0.6 μM: 0.6 μM. The modified Triplet-repeat Primed PCR was performed in a 25 μl volume using the FailSafe^TM^ PCR system (Madison, WI 53719, USA). The reactions were subjected to 1 cycle of 95°C for 5 min, and 10 cycles of 97°C for 35 s, 65°C for 35 s and 68°C for 4 min, followed by 20 cycles in which the extension time was increased by 20 s per cycle to allow for increased yield of PCR product. TP-PCR was repeated with Hex labeled P2-Reverse to rule out any false negative results from CAG interruptions. The sequence information of the TP-PCR primers is listed in **Table 1**.

**Table 1:** Triplet-Primed PCR primer sequences.

	Primer name	Primer sequence
Forward direction TP-PCR	FAM-P1-forward	5′FAM-GGG-GCT-CGA-AGG-GTC-CTT-GT-3′
	P4-(CAG)_6_-reverse	5′AGC-GGA-TAA-CAA-TTT-CAC-ACA-GGA-CAG-CAG-CAG-CAG-CAG-CAG-3′
	P3	5′AGC-GGA-TAA-CAA-TTT-CAC-ACA-GGA- 3′
	P2-reverse	5′-GTG-CGT-GGA-GGA-TGG-AAC-ACG-3′
Reverse direction TP-PCR	P1-forward	5′-GGG-GCT-CGA-AGG-GTC-CTT-GT-3′
	P4(CTG)6-forward	5′AGA-GGA-TAA-CAA-TTT-CAC-ACA-GGA-TGC-TGC-TGC-TGC-TGC-TGC-TG-3′
	P3	5′AGC-GGA-TAA-CAA-TTT-CAC-ACA-GGA- 3′
	Hex-P2-reverse	5′Hex-GTG-CGT-GGA-GGA-TGG-AAC-ACG-3′

### PCR PRODUCT ANALYSIS ON GENETIC ANALYZER

Products were separated on an ABI PRISM 3130 × l genetic analyzer (Life Tech, Grand Island, NY 14072, USA). 1 μl of PCR product was mixed with 0.5 μl of MapMarker ROX 1000 (Bioventures, Murfreesboro, TN, USA) and 9 μl of HiDi Formamide (Life Tech, Grand Island, NY 14072, USA). The mixture was denatured at 95°C for 5 min then loaded onto the 3130 × l genetic analyzer. Fragment size was analyzed using GeneMarker V2.6 (Softgenetics, State College, PA, USA).

### SOUTHERN BLOT

With the purpose to confirm and measure the abnormal alleles, Southern Blots tests were performed in the samples indicating one normal allele by PCR. 47 μg of genomic DNA was digested with Bgl 1 restriction enzyme. The digested DNA was separated on a 1% agarose gel at 60 volts for 18 h. The gel was depurinated, denatured, neutralized, and DNA was transferred to a positive nitrocellulose membrane (Biodyne B 0.45UM, VWR, PA 19087, USA). After transfer, the membrane was hybridized with ^32^P labeled DM1 probe and exposed with X-ray film (Kodak, Rochester, NY, USA) overnight or longer as appropriate.

## RESULTS

Forty five samples had been previously tested with conventional PCR and reflex Southern blot. Of those 45, 24 gave two distinct allele peaks by PCR. 23 of the 24 samples had two normal alleles and one sample showed one normal allele and one abnormal allele with a CTG repeat size of 85 repeats. The 85 repeat allele is the largest detected by conventional PCR and sizable by TP-PCR. 21 patients had only one peak with the repeat size falling in the normal range. Southern Blot was performed as a reflex test and an expanded band was detected in 13 of the 21 samples. These large alleles were missed by conventional PCR and ranged from 150 CTG repeats to 1700 repeats.

**FIGURE 2:**
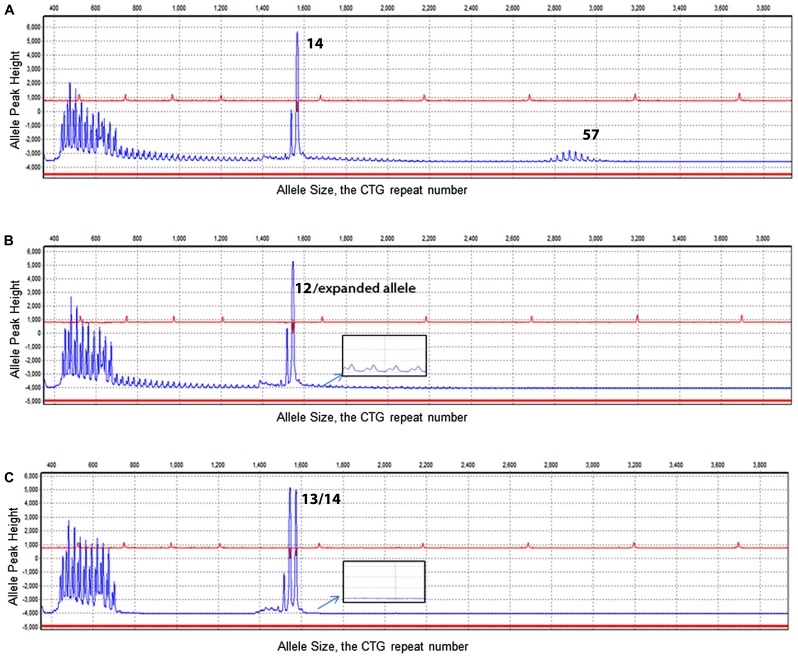
**Electropherogram results of TP-PCR**. X-axis illustrates the CTG repeat number. Y-axis illustrates the allele peak height. **(A)** Electropherogram of DM1 genotyping of a sample showing alleles of 14/57 CTG repeats. **(B)** Electropherogram result of a sample with a normal allele was determined with a size of 12 CTG repeats. In addition to the normal allele, a ladder signal was represented by TP-PCR indicating the existence of an expanded allele (see enlarged insert). **(C)** Demonstrates two normal heterozygous alleles (13/14) and no laddering signal from the TP-PCR (see enlarged insert).

To validate an efficient clinical technique to detect the presence of the large alleles and reduce the use of the Southern Blot test, a modified TP-PCR was utilized with the same sample set. In this study, the TP-PCR was modified from a two-tube reaction to a one-tube reaction. With a combination of one set of gene specific primers and one hybrid triplet primer, the TP-PCR is capable of amplifying normal alleles and screening expanded alleles in one tube reaction. The normal allele sizes from the TP-PCR indicated good concordance with the conventional PCR (**Table 2**). Of 13 abnormal samples missed by conventional PCR, a ladder pattern was found indicating the presence of an expanded allele (**Figure 2**). Thus, by screening with TP-PCR, only 13 of 21 samples that had one allele with conventional PCR would need the Southern Blot test. The need for the reflex test is reduced by 38.1%. The detection rate for the abnormal alleles is 100%. In order to rule out false negative results due to the CCG, CTC, or GGC interruptions, TP-PCR was also performed with a HEX- labeled P2-Reverse primer. The P2-Reverse primer gave a consistent, one triplet repeat smaller size than that indicated by the FAM-labeled TP-PCR (**Table 2**).

**Table 2:** CTG size information obtained from Triplet-repeat Primed PCR, conventional PCR and Southern Blot.

	Results from Triplet Primed PCR			Results from conventional PCR and Southern Blot	
Sample ID	CTG number, FAM labeled-forward primer	CTG number, HEX labeled-reverse primer	Detection of expanded allele	CTG number, FAM labeled-forward primer	CTG number, convention PCR with reflex Southern Blot
DM-1	5	4	No	5	5
DM-2	5/85*	4/84*	Yes	5/85*	5/85*
DM-3	5	4	Yes	5	5/700*
DM-4	11/12	10/11	No	11/12	11/12
DM-5	13/14	12/13	No	13/14	13/14
DM-6	12	11	No	12	12
DM-7	13	12	No	13	13
DM-8	13	12	No	13	13
DM-9	21	20	No	20	20
DM-10	12/13	11/12	No	12/13	12/13
DM-11	5	4	Yes	5	5/1300–1700*
DM-12	12/13	11/12	No	11/12	11/12
DM-13	12	11	Yes	11	11/700–1700*
DM-14	14	13	Yes	13	13/1800*
DM-15	5	4	Yes	4	4/150–450*
DM-16	11/12	10/11	No	10/11	10/11
DM-17	11/13	10/12	No	11/12	11/12
DM-18	12/13	11/12	No	10/11	10/11
DM-19	5	4	No	4	4
DM-20	10/16	9/15	No	10/16	10/16
DM-21	12	11	Yes	12	12/350*
DM-22	5/12	4/11	No	5/12	5/12
DM-23	12	11	Yes	12	12/550*
DM-24	12/13	11/12	No	12/13	12/13
DM-25	10/16	9/15	No	10/16	10/16
DM-26	21	20	Yes	21	21
DM-27	5/13	4/12	No	5/13	5/13
DM-28	12/13	11/12	No	12/13	12/13
DM-29	12	11	Yes	11	11/380*
DM-30	5/11	4/10	No	5/10	5/10
DM-31	5/12	4/11	No	4/11	4/11
DM-32	5/13	4/12	No	4/12	4/12
DM-33	12	11	Yes	11	11/600*
DM-34	5/13	4/12	No	4/12	4/12
DM-35	10/16	9/15	No	9/15	9/15
DM-36	12/13	11/12	No	11/12	11/12
DM-37	12	11	Yes	11	11/350*
DM-38	10/16	9/15	No	9/15	9/15
DM-39	13/20	12/19	No	12/19	12/19
DM-40	12/14	11/13	No	11/13	11/13
DM-41	12	11	Yes	11	11/409*
DM-42	12	11	Yes	11	11/350*
DM-43	5/11	4/10	No	4/10	4/10
DM-44	5	4	No	4	4
DM-45	12	11	Yes	11	11/634*

* indicates an expanded allele.

## DISCUSSION

Clinical testing of the CTG expansion remains a challenge due to the large repeat size, especially in classical or congenital DM1 patients. The traditional method applied in the molecular diagnosis of DM1 is currently conventional PCR in conjunction with Southern Blot to detect and measure the large expansions. Conventional PCR is used as the first step to determine alleles with a low number of repeats. Due to the limitations of conventional PCR, true homozygous normal patients cannot be differentiated from the heterozygous patients with one normal and one large expanded allele. Thus, if only one allele is amplified from conventional PCR, a subsequent test, Southern Blot, is used to test for possible larger expansions. This makes the molecular diagnosis of DM1 complicated and time consuming.

TP-PCR was first introduced by Warner et al. (1996) and was advantageous in the reduced need to perform Southern Blot. With TP-PCR, the true homozygotes and false negative homozygotes can be distinguished based on the presence or absence of an expanded triplet ladder pattern. After TP-PCR screening, only patients displaying a laddering tail will receive a reflex Southern blot test. It significantly reduces the need of the Southern Blot test. In our study, the application of TP-PCR decreased this need by 38.1%. However, the previous reported TP-PCR cannot provide genotyping information. An additional regular PCR step is required to size the normal and small expanded alleles. The genotyping of the large alleles still has to rely on Southern blot.

An improved TP-PCR had been reported in the molecular diagnosis of Fragile X (Tassone et al., 2008; Chen et al., 2010). They developed a single-tube CGG repeat primed *FMR1* PCR test that can detect the full range of *FMR1* expanded alleles. This method includes one set of gene specific primers plus one chimeric PCR primer that targets randomly within the expanded CGG region. According to their report, the method is able to size expansions/alleles up to 200 CGG repeats and detect full-mutation ranges from one single test. In our studies, in order to simplify the testing procedure and reduce the cost, we combined the conventional fluorescent PCR and TP-PCR into a one-tube multiplex PCR. A second gene specific primer pairing to P1 was introduced at an optimized ratio that allowed the amplification of the normal and mutable alleles. The modified TP-PCR is still able to detect the expanded mutants and size the normal or premutated alleles in one single reaction (**Figure 2**). Therefore, conventional PCR is not required to size the normal alleles in the DM1 patients.

The presence of CCG interruptions could potentially lead to the drop-out of the abnormal allele from TP-PCR (Santoro et al., 2013). In order to avoid the false negative results due to CCG interruptions, TP-PCR is recommended to be performed in both directions with two different fluorescent dyes. In our study, the TP-PCR was performed from both the forward and reverse directions in 45 samples (FAM TP-PCR and HEX TP-PCR). The testing results demonstrated good concordance with previous results for the 45 samples. Alleles sized by the HEX TP-PCR consistently displayed one CTG repeat smaller than the FAM labeled amplicons. This size difference is reported to be from the mobility shift variance resulting from the structure of fluorescent dyes and different composition of the single-stranded DNA (Radvansky et al., 2011). All eight samples that were indicated to be normal homozygotes with FAM TP-PCR were also indicated to be normal homozygotes with HEX TP-PCR. This helps rule out false negatives generated from CCG interruptions.

In conclusion, the modified bidirectional TP-PCR test is capable of differentiating normal, premutated, and fully mutated alleles in *DMPK*. It improves the traditional TP-PCR by enabling reduction from two PCR procedures to one PCR procedure. The modified TP-PCR can accurately detect the presence of the large expanded allele and significantly reduce the need of the Southern Blot test. It is simple, sensitive, and reliable for clinical testing of DM1 patients and can be an efficient screening method in the clinical diagnosis of myotonic dystrophy type 1.

## AUTHOR CONTRIBUTIONS

Shaochun Bai, Susmita Singh, and Stephen Dlouhy designed the study. Susmita Singh and Amy Zhang procured samples and reagents. Susmita Singh analyzed the data. All authors contributed to the writing and proof reading of this manuscript.

## Conflict of Interest Statement

The authors declare that the research was conducted in the absence of any commercial or financial relationships that could be construed as a potential conflict of interest.

## References

[B1] Bird T. (2013). *Myotonic Dystrophy Type 1. GeneReviews*. Available at: http://www.ncbi.nlm.nih.gov/books/NBK1165/

[B2] Chen L. Hadd A. Sah S. Filipovic-Sadic S. Krosting J. Sekinger E. (2010). An information-rich CGG repeat primed PCR that detects the full range of fragile X expanded alleles and minimizes the need for southern blot analysis. *J. Mol. Diagn.* 12 589–600 10.2353/jmoldx.2010.090227 20616364 PMC2928422

[B3] Gharehbaghi-Schnell E. B. Finsterer J. Korschineck I. Mamoli B. Binder B. R. (1998). Genotype – phenotype correlation in myotonic dystrophy. *Clin. Genet.* 53 20–26 10.1034/j.1399-0004.1998.531530105.x 9550357

[B4] Johnson N. E. Heatwole C. R. (2012). Myotonic dystrophy: from bench to bedside. *Semin. Neurol.* 32 246–254 10.1055/s-0032-1329202 23117949

[B5] Prior T. W. American College of Medical Genetics (ACMG) Laboratory Quality Assurance Committee. (2009). Technical standards and guidelines for myotonic dystrophy type 1 testing. *Genet. Med*. 11 552–555 10.1097/GIM.0b013e3181abce0f 19546810

[B6] Radvansky J. Ficek A. Minarik G. Palffy R. Kadasi L. (2011). Effect of unexpected sequence interruptions to conventional PCR and repeat primed PCR in myotonic dystrophy type 1 testing. *Diagn. Mol. Pathol.* 20 48–51 10.1097/PDM.0b013e3181efe290 21326039

[B7] Santoro M. Masciullo M. Pietrobono R. Conte G. Modoni A. Bianchi M. L. (2013). Molecular, clinical, and muscle studies in myotonic dystrophy type 1 (DM1) associated with novel variant CCG expansions. *J. Neurol.* 260 1245–1257 10.1007/s00415-012-6779-9 23263591

[B8] Tassone F. Pan R. Amiri K. Taylor A. K. Hagerman P. J. (2008). A rapid polymerase chain reaction-based screening method for identification of all expanded alleles of the fragileX (FMR1) gene in newborn and high-risk populations. *J. Mol. Diagn.* 10 43–49 10.2353/jmoldx.2008.070073 18165273 PMC2175542

[B9] Turner C. Hilton-Jones D. (2010). The myotonic dystrophies: diagnosis and management. *J. Neurol. Neurosurg. Psychiatry* 81 358–367 10.1136/jnnp.2008.158261 20176601

[B10] Warner J. P. Barron L. H. Goudie D. Kelly K. Dow D. Fitzpatrick D. R. (1996). A general method for the detection of large CAG repeat expansions by fluorescent PCR. *J. Med. Genet*. 33 1022–1026 10.1136/jmg.33.12.1022 9004136 PMC1050815

[B11] Zeesman S. Carson N. Whelan D. T. (2002). Paternal transmission of the congenital form of myotonic dystrophy type 1: a new case and review of the literature. *Am. J. Med. Genet*. 107 222–226 10.1002/ajmg.10141 11807903

